# Genome-wide prediction of prokaryotic two-component system networks using a sequence-based meta-predictor

**DOI:** 10.1186/s12859-015-0741-7

**Published:** 2015-09-18

**Authors:** Altan Kara, Martin Vickers, Martin Swain, David E. Whitworth, Narcis Fernandez-Fuentes

**Affiliations:** Institute of Biological, Environmental and Rural Sciences, Aberystwyth University, Aberystwyth, SY23 3EB UK

**Keywords:** Two-component signalling system, Protein-protein interactions, Protein-protein interaction predictions, Meta-predictor, Support vector machine, Web server, Genome context, Co-evolution

## Abstract

**Background:**

Two component systems (TCS) are signalling complexes manifested by a histidine kinase (receptor) and a response regulator (effector). They are the most abundant signalling pathways in prokaryotes and control a wide range of biological processes. The pairing of these two components is highly specific, often requiring costly and time-consuming experimental characterisation. Therefore, there is considerable interest in developing accurate prediction tools to lessen the burden of experimental work and cope with the ever-increasing amount of genomic information.

**Results:**

We present a novel meta-predictor, MetaPred2CS, which is based on a support vector machine. MetaPred2CS integrates six sequence-based prediction methods: *in-silico* two-hybrid, mirror-tree, gene fusion, phylogenetic profiling, gene neighbourhood, and gene operon. To benchmark MetaPred2CS, we also compiled a novel high-quality training dataset of experimentally deduced TCS protein pairs for k-fold cross validation, to act as a gold standard for TCS partnership predictions. Combining individual predictions using MetaPred2CS improved performance when compared to the individual methods and in comparison with a current state-of-the-art meta-predictor.

**Conclusion:**

We have developed MetaPred2CS, a support vector machine-based metapredictor for prokaryotic TCS protein pairings. Central to the success of MetaPred2CS is a strategy of integrating individual predictors that improves the overall prediction accuracy, with the *in-silico* two-hybrid method contributing most to performance. MetaPred2CS outperformed other available systems in our benchmark tests, and is available online at http://metapred2cs.ibers.aber.ac.uk, along with our gold standard dataset of TCS interaction pairs.

**Electronic supplementary material:**

The online version of this article (doi:10.1186/s12859-015-0741-7) contains supplementary material, which is available to authorized users.

## Background

A wide range of critical functions in prokaryotes such as antibiotic resistance, stationary phase transition, competence, sporulation, chemotaxis, nitrogen regulation, virulence, and phosphate regulation are mediated by a particular type of signalling pathway known as a two-component system (TCS) [1]. TCS typically operate through the transfer of phosphoryl groups from a His residue of a histidine kinase (HK) to an Asp residue of a response regulator (RR), in response to an extracellular stimulus. A variant of the TCS, known as a phosphorelay, includes extra receiver and phosphotransfer domains relaying the phosphoryl group between the HK and RR proteins [2].

Genome-wide identification of HK and RR proteins is relatively straightforward [3], with a variety of TCS databases and prediction servers available [4–6]. However the identification of HK-RR pairs is challenging as TCS pairs are highly specific [7], there are multiple HK and RRs in most genomes, and their genes are often unpaired (orphan HKs and RRs). Several experimental approaches have been used to identify HK-RR pairs, including phosphotransfer profiling [8–10] and yeast two-hybrid assays [11–14]. Such approaches are costly and labour intensive, therefore it is important to develop computational tools to lessen the burden and complement experimental approaches.

The use of meta-predictors, predictors that combine predictions from individual methods using machine learning algorithms, is a common approach in bioinformatics [15–21]. The advantage of meta-predictors is that they do not rely on single methods, but can integrate a wide range of information under a common probabilistic umbrella without relying on complex scoring functions [22]. In doing so, the strengths and weaknesses of individual predictions are combined to achieve higher levels of accuracy [21]. Examples of meta-predictors include those developed for the prediction of functional sites in proteins [23] or prediction of critical residues in protein interfaces [24].

In this work we present MetaPred2CS, a sequence-based meta-predictor designed specifically to predict protein pairs in TCS. MetaPred2CS is based on a Support Vector Machine (SVM) [25, 26] and combines six independent and orthologous protein-protein interaction prediction methods: in-silico two-hybrid (i2h) [27], mirror tree (MT) [28], phylogenetic profiling (PP) [29], gene fusion (GF) [30], gene neighbourhood (GN) and gene operon (GO) [31]. The i2h and MT methods are based on co-evolution theory, and rely on high quality and complete multiple sequence alignments (MSAs), while PP, GF, GN and GO, are genome context methods, utilising different genomic information such as chromosomal proximity (GN), operons (GO), fusion events (GF) and inter-genomic profiles (PP) between fully sequenced genomes.

The identification performance of MetaPred2CS was tested using validated experimental data and it achieved a higher accuracy compared to individual prediction methods such as i2h, MT, GF, PP, GN and GO. MetaPred2CS also compared favourably against a Bayesian meta-predictor benchmarked on TCS pairs [32] and a database of protein-protein interactions: STRING [33].

## Methods

### Datasets: training and testing

A variety of datasets, described in detail below, were used during the development of the predictor to benchmark its performance under different scenarios and to compare to an independent, competing, method and pre computed scores in the STRING [33] database. File1.xls and File2.pdf in the Additional files 1 and 2 provide complete details and a diagrammatic representation of the all sets described below.

#### The P+ and P- datasets

The P+ and P- sets contain 113 interacting and 1134 non-interacting experimentally validated TCS pairs respectively, and were compiled and manually curated from the current literature. These sets were used to train and test the MetaPred2CS using a k-fold cross validation strategy. Specifically, the P+ set was compiled by mining protein-protein interaction databases, including BioGriD [34], DIP [35], IntAct [36], PSI-MI [37], UniProtKB [38] and MINT [39] using the RefSeq identifiers extracted from the P2CS database [4]. To create P-, experimentally validated non-interacting pairs were mined from publications describing high-throughput, systematic, yeast two-hybrid or phosphotransfer profiling experiments, from a number of organisms including: *Caulobacter crescentus*, *Escherichia coli*, *Mycobacterium tuberculosis*, *Myxococcus xanthus*, *Synechocystis sp.* and *Mesorhizobium loti* [8–14].

To test the performance of MetaPred2CS under different scenarios and to compare it to an independent, competing, methods, we derived interdependent testing sets as described below (NP+, OP+, species-specific, T, SP+ and SP- sets). In each test, MetaPred2CS was trained with the corresponding, orthogonal, version of P+ and P- (i.e. removing any proteins present in the testing subset).

#### NP+ and OP+ and Species-specific datasets

The NP+ set (for Neighbouring Pairs) contains 56 pairs of TCS that are encoded by neighbouring genes, while the OP+ set (for Orphan Pairs) is composed of 57 pairs that are encoded by genes, which are not adjacent in the genome. This distinction is important, as predictions of orphan pairs are usually more challenging [40–42]. In order to further clarify species-specific and positive-to-negative class ratio bias in the predictions, we also produced four different species-specific testing sets: *Escherichia coli, Myxococcus xanthus, Synechocytis sp, and Mesorhizobim lotis*.

#### T, SP+ and SP- datasets

Datasets T, SP+ and SP- were extracted from the work by Burger and van Nimwegen [32] as testing sets to compare MetaPred2CS performance. For all these three testing sets, MetaPred2CS was trained with an orthogonal version of P+ and P- sets, i.e. any pair present in either of the testing sets was removed from P+ or P- prior to training. The T dataset is composed of 16 experimentally validated interacting pairs and 5 non-interacting pairs while the SP+ and SP- sets are composed of pairs of TCS extracted from the SwissRegulon database [32]. In addition, The SP+ and SP- was also used to compare to STRING [33] database.

### The MetaPred2CS prediction method

#### Individual prediction methods

The selection of individual methodologies was based on their orthogonalitynature, i.e. sequence-based, performance and availability. MetaPred2CS integrates the prediction of six different methods: i2h, MT, PP, GF, GN and GO. Briefly, the i2h method scans for correlated (compensatory) mutations between residues of the two proteins of interest, where a high-correlation implies high-probability that given pair interacts [27]. The MT method relies on similarity between phylogenetic trees to infer the likelihood of interactions between pairs of proteins [28]. The GN and GO methods are based on the observation that proteins that are functionally related tend to be transcribed and expressed concurrently, i.e. are encoded by adjacent genes, particularly in prokaryotes [31]. The PP method is based on the idea that functionally related genes under strong selective pressure appear or disappear together as units during speciation events [29]. Finally, the GF method is based on fusion events, i.e. if two proteins appear as independent units in one organism but as a joint entity in another organism, then it is likely that the individual units are actually an interacting pair [30]. Detailed information about these methods as well as their technical aspects can be found in the individual references indicated above.

#### Reference genome dataset

The GN, GO, GF, and PP methods rely on a reference genome dataset, the quality of which, in the form of size and diversity, is central to their performance [29]. To that end, and to maximize the prediction performance of these methodologies, we compiled a diverse, yet relatively small (as a compromise between performance and calculation speed), reference genome dataset based on the most successful genome combinations proposed by Muley and Ranjan [43]. Our dataset comprised 243 individual genomes, belonging to 22 different classes (for further details see Table S1 in the Additional file 3). Genome annotations were downloaded from the NCBI database [44] and operon data from annotated genomes using Moreno-Hagelsieb and Collado-Vides' approach [45] available at http://microbiome.wlu.ca/public/TUpredictions/Predictions/ .

#### Implementation of individual prediction methods

All methods were implemented as described in their original publications. Co-evolutionary-based methods (i2h and MT) rely on the quality of MSAs, i.e. their completeness and diversity. We generated MSAs following the approach described in our previous work [46], using UniprotKB [38]. ParseBlast generates complete and diverse MSAs by filtering both highly identical and highly dissimilar sequence homologs, considering also the coverage of the alignment between query and hit proteins [46]. With the exception of the minimum and maximum number of species represented in the MSAs, 25 and 50 respectively, the rest of the prediction parameters were set to default values as described in the original works [27, 28]. These two parameters set the number of sequences shared between both MSAs, which include the sequences of common species in both alignments selecting the ones with the highest sequence identity to the corresponding pair. Thus, the minimum and maximum number in common between the two MSAs is an important aspect on these methodologies as its performance is highly influenced by these two parameters, i.e. the diversity of alignment.

We used InPrePPI [47], which implements all the genome context based methods (GF, PP, GN and GO), which requires a reference genome dataset and genome annotation (e.g. operon units) as described above. Among the parameters required for prediction are: (i) the evolutionary distances between target and reference organisms, which was calculated using 16S RNA data as described previously [47]; (ii) an e-value cut-off of 1e-5 for BLASTP [48] searches; (iii) a cut-off of 0.35 for mutual information values (required for PP); and (iv) a distance cut-off of 200 bp for the GN/GO predictions, as suggested previously [45, 49, 50]. The GF method identified fusions events, which in the case of TCS result in hybrid proteins combining a HK and RR in single coding unit, by using local alignments based on the Smith-Waterman algorithm [51], implemented in the ssearch36 program using default parameters [52].

#### Integration of individual prediction methods using a SVM: MetaPred2CS

**Fig. 1:**
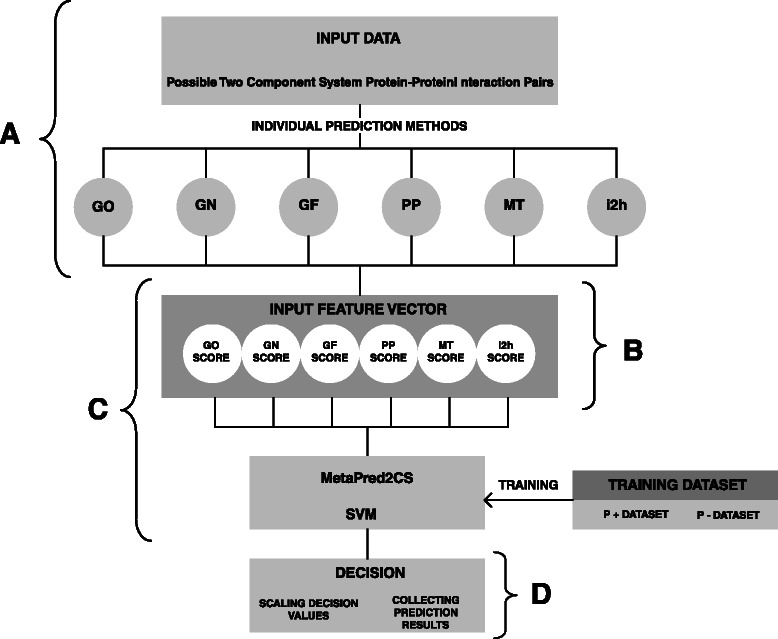
Schematic representation of MetaPred2CS. Individual predictions are performed for given pairs of HK and RR (**a**). The prediction scores are then used as the input vector for the SVM (**b**) trained in the P+ and P- sets (**c**). Finally, prediction scores are scaled from 0 to 1 (**d**)

MetaPred2CS is based on a support vector machine (SVM), implemented using the LIBSVM package [53]. The individual prediction methods described above form a six-dimensional vector representing the prediction scores for a given pair of proteins of interest, i.e. a HK and RR pair. The vector is then inputted into a SVM trained using the same training set. The -*w* option in LIBSVM was used to account for the imbalance between positive and negative classes. Also, the optimal values for the error cost (c) and the gamma value (g), were explored using a grid search on a 10-fold cross validation with the radial basis kernel function [54] (see Table S2 in the Additional file 3). Finally, decision values were normalized in a range between 0 and 1 (Fig. 1).

#### Benchmarking and comparison of MetaPred2CS performance

MetaPred2CS was benchmarked and assessed using different datasets (described above). Firstly, to assess the contribution of each individual prediction method to the final classifier, we trained and test 8 different classifiers with different combinations of individual prediction methods (Table 1). Each of these different classifiers was assessed using 5-, 10- and 20-fold cross-validation using the P+ and P- datasets. Furthermore, MetaPred2CS, was benchmarked against NP+, OP+, and specie-specific testing sets were used to further discern the performance of predictions in orphans and neighbouring genes and specie-specific sets. Finally, MetaPredCS was compared against the work by Burger and van Nimwegen [32] (T, SP+ and SP- datasets) and STRING [33] database (SP+ and SP- datasets).

**Table 1:** AUC values of predictions by individual methods for the P+/P-, NP+/P- and OP+/P- datasets. GN and GO methods were not included the AUC comparison given the large genomic distance between pairs on the P- dataset that made the predictions unfeasible

Datasets	AUC Values of Individual Methods					
	i2h	MT	GF	PP	GN	GO
P+/P-	0.84	0.66	0.58	0.57	N/A	N/A
NP+/P-	0.90	0.69	0.60	0.55	N/A	N/A
OP+/P-	0.78	0.63	0.55	0.59	N/A	N/A

#### Assessing MetaPred2CS performance

The performance of each classifier was evaluated according to sensitivity (1), specificity (2), accuracy (3), Mathew’s correlation coefficient MCC [55] (4) and Area Under the ROC Curve (AUC) [56] values. Formally,

1 $$ Sensitivity=\frac{TP}{TP+FN} $$

2 $$ Specificity=\frac{TN}{TN+FP} $$

3 $$ Accuracy=\frac{TP+TN}{TP+TN+FP+FN} $$

4 $$ MCC=\frac{TP\times TN-FP\times FN}{\sqrt{\left(TP+FP\right)\left(TP+FN\right)\left(TN+FP\right)\left(TN+FN\right)}} $$

Where TP, FP, TN, and FN represent true positives, false positives, true negatives and false negatives, respectively. Particularly important are the MCC values, given the disequilibrium of positive and negative classes, i.e. the difference in size of interacting and non-interacting pairs. The statistical analysis of ROC curves was performed using STAR [57].

## Results and Discussion

### Evaluation of individual prediction methods

Individual methods were tested on the P+/P-, NP+/P-, and OP+/P- datasets. Prediction performance metrics of each method are presented and compared using AUC values (Table 1). Co-evolutionary methods (i2h and MT) performed better than the genomic context methods, and the i2h method outperformed all other methods for each dataset, with MT being the next best method for each dataset.

With the exception of the PP, the best performance was on the NP+/P- dataset. This was expected because predicting orphan pairs is usually more challenging than neighboring pairs. PP however rely on comparison across genomes where interacting pairs either appear or disappear concurrently, hence genomic context does not play a unique contribution. Consequently, PP achieved the best performance on the OP+/P- dataset of the genomic context methods. It also performed similarly to the GF method on the P+/P- dataset. In the case of GN and GO, intrinsic limitations of these methodologies, i.e. rely on genomic distance, prevented its use on the P- and OP+ dataset, hence AUC and MCC could not be calculated, hence not presented in Table 1. Nonetheless, GN and GO are valid strategies in the prediction of pairing in neighboring genes (51 pairs out of 57 on the NP+ were predicted correctly), hence GH and GO predictions were considered as part input vector for the meta-predictor (see next).

### Contribution of individual methods to MetaPred2CS

To understand the contribution of individual methods, several meta-predictors were trained and tested using different K-fold cross-validation strategies on the P+/P- sets. The different combinations of individual predictors are listed in Table 2. The meta-predictor combining all six-prediction methods, hereinafter referred to as the default predictor or MetaPred2CS, achieved the highest performance (AUC: 94.79; MCC: 0.51). The largest drop in performance resulted when the i2h method was removed from the input features vector (AUC: 88.87; MCC: 0.401). Omitting a method had a minimal effect if another method(s) based on similar principles, e.g. genomic-context, was retained. For example, when GO was excluded but GN was kept, the decreases in AUC and MCC were very small (AUC: 94.76; MCC: 0.46). However, when excluding both GN and GO methods together, the decrease in AUC and MCC was larger (AUC: 89.83; MCC: 0.41). Tests performed at different cross-validation levels also showed that the different sizes of the training and test datasets did not result in a large differences in the performance of SVM classifiers with our training dataset and the best results were obtained at 10-fold cross-validation (Table S2 Additional file 3).

**Table 2:** Combinations of prediction methods and prediction performance at 10-fold cross-validation. 1: i2h not included, 2: MT not included, 3: GF not included, 4: PP not included, 5: GN not included, 6: GO not included, 7: GN and GO not included, 8: all methods included. AUC and MCC represent the area under the ROC curved and Matthew’s correlation coefficient respectively

Combinations	AUC Values	MCC Values
1 : i2h method excluded	88.86	0.401
2 : MT method excluded	94.69	0.500
3 : GF method excluded	94.45	0.484
4 : PP method excluded	91.89	0.414
5 : GN method excluded	94.04	0.454
6 : GO method excluded	94.76	0.504
7 : GN/GO methods excluded	90.15	0.408
8 : all methods included	94.79	0.508

### Species-specific predictions

To further characterise the performance of MetaPred2CS, we performed species-specific predictions. Four independent testing sets representing *Escherichia coli, Myxococcus xanthus, Synechocytis sp, and Mesorhizobim loti* were created, due to the number of TCS proteins encoded in their genomes and the resulting ratio between interacting and non-interacting pairs. As shown in several works (e.g. [58, 59]) the ratio between positive and negative cases has an important impact in the performance of predictors of protein-protein interactions. *Escherichia coli* represents the organism with the lowest number of TCS proteins (62) and the lowest interacting (22) to non-interacting (64) pairs ratio (approximately 1:3) while *Myxococcus xanthus* had the 236 pairs and a ratio of 20:216 interactiong:non-interacting pairs. The most challenging cases were *Synechocystis sp.* and *Mesorhizobium loti* with 20 interacting to 319 non-interacting pairs and 20 interacting to 364 non-interacting pairs, respectively. Overall and as expected, the best prediction performance was achieved for *Escherichia coli*, although predictions were still accurate even for *Synechocystis sp.* and *Mesorhizobium loti* (Table 3).

**Table 3:** Performance of default predictor on species-specific gene sets. Sensitivity, specificity, accuracy and MCC values are presented, as defined in the text

Species used as test data	Performance of Classifier			
	Sensitivity	Specificity	Accuracy	MCC
*Escherichia coli* K-12 MG1655	0.82	0.86	0.85	0.607
*Myxococcus xanthus* DK1622	0.92	0.87	0.87	0.582
*Synechocystis sp*. PCC6803	0.81	0.86	0.77	0.477
*Mesorhizobium loti* MAFF303099	0.75	0.89	0.88	0.476

### Predictions of neighbouring and orphan pairs (NP+/P- and OP+/P- sets)

**Fig. 2:**
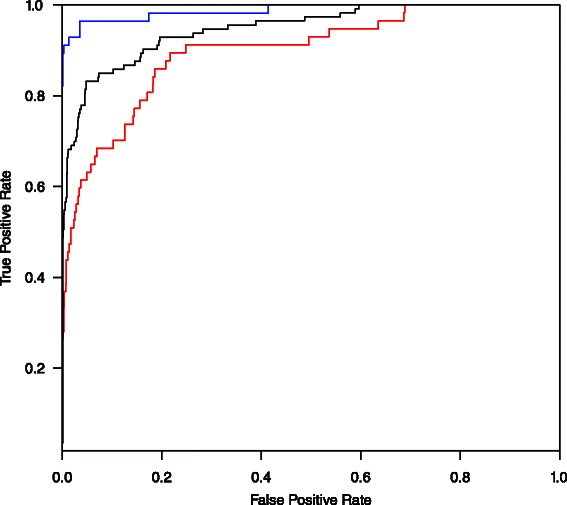
ROC curves of predictions on the NP+/P-, P+/P- and OP+/P- datasets using default predictor. Blue, black and red ROC curves represent the performance on the NP+/P-, P+/P- and OP+/P- datasets, respectively

The genes encoding a TCS pairs can be located in adjacent (neighbouring) or separate (orphan) positions within the genome. The prediction of interacting pairs would be expected to be more challenging for orphans than for neighbouring proteins. Therefore, to test the capacity and performance of MetaPred2CS under these different scenarios, the P+ dataset was divided into two subsets: NP+ (neighbouring pairs) and OP+ (orphan pairs), and assessed at different K-fold cross validations. As expected, MetaPred2CS performed better on the NP+/P- than on the OP+/P- set at any K-fold validation values (Table 4). The best performance was achieved at the 10-fold cross-validation level. ROC curves of NP+/P- (AUC = 0.98), P+/P- (AUC = 0.95) and OP+/P- (AUC = 0.89) datasets at 10-fold cross-validation are shown in Fig. 2.

**Table 4:** Prediction performance of default predictor on neighbouring and orphan pairs. AUC and MCC values for MetaPred2CS trained on the NP+/P- and OP+/P- datasets at different level of K-fold cross-validation

Dataset	Performance of Classifier According to Cross-validation Levels					
	5-fold		10-fold		20-fold	
	AUC	MCC	AUC	MCC	AUC	MCC
NP+/P-	98.79	0.639	98.40	0.639	98.75	0.634
OP+/P-	90.28	0.409	89.36	0.407	90.31	0.410

### Comparison of MetaPred2CS and a competing machine-learning method and STRING database

MetaPred2CS was compared to a competing machined learning method publicly available using common testing sets [32] and STRING [33] database. On the first instance, both methods were compared using the T set compiled in Burger and van Nimwegen’s original work [32]. The T set is composed of 16 interacting and 5 non-interacting protein pairs. As shown in Table 5, out of 21 pairs, 16 were predicted more accurately by MetaPred2CS (4 cases both methods performed at the same level). Moreover, MetaPred2CS correctly predicted all non-interacting pairs, assigning low prediction scores for all cases. The T dataset is however a small set composed of protein pairs from a single specie: *Caulobacter crescentus*.

**Table 5:** Prediction of the T dataset by the Bayesian approach [32] and MetaPred2CSc. Non-interacting protein pairs are marked with an asterisk and best predictions are highlighted in bold

Type^(a)^	Protein Pairs	Bayesian Approach	MetaPred2CS
IT	CC0248 - CC0247	**1.000**	0.894
IT	CC0289 - CC0294	**0.995**	0.633
IT	CC2755 - CC2757	**0.851**	0.164
IT	CC2765 - CC2766	**1.000**	0.852
IT	CC2932 - CC2931	0.945	**1.000**
IT	CenK - CenR	**0.917**	0.491
IT	CckN - DivK	0.306	**0.649**
IT	ChpT - CtrA	0.197	**0.786**
IT	ChpT - CpdR	0.001	**0.650**
IT	DivJ - CtrA	0.461	**0.559**
IT	DivJ - PleD	0.385	**0.723**
IT	DivJ - DivK	0.041	**0.756**
IT	DivL - DivK	0.537	**0.559**
IT	DivL - CtrA	0.130	**0.721**
IT	PleC - DivK	0.080	**0.477**
IT	PleC - PleD	0.001	**0.600**
NI	ChpT - CC3477*	0.607	**0.231**
NI	ChpT - CC2757*	0.128	**0.000**
NI	ChpT - CenR*	0.067	**0.000**
NI	PleC - CtrA*	**0.002**	0.022
NI	PleC - CC3477*	0.001	**0.000**

^(a)^IT: interacting pair; NI: non-interacting pair

**Fig. 3:**
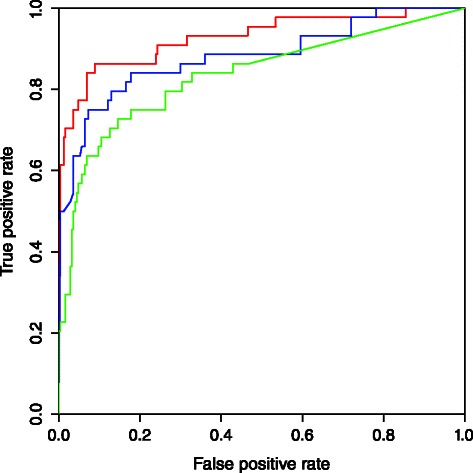
ROC curves of predictions on the SP+/SP- datasets. Red, blue and green ROC curves represent predictions by MetaPred2CS, STRING [33], and the Bayesian approach of Burger and van Nimwegen [21], respectively

A more comprehensive comparison was carried out on the SP+/SP- dataset, also compiled Burger and van Nimwegen’s original work [32], which is considerably larger and more diverse, comparing also to STRING [33] database. These datasets include protein pairs from 6 different species: *Escherichia coli*, *Bacillus subtilis*, *Caulobacter crescentus*, *Mesorhizobium loti*,* Myxococcus xanthus*, and *Synechocystis sp.* As shown in Fig. 3, MetaPred2CS performed better than the Bayesian approach (AUC: 92.8 vs. 83.5) and STRING [33] database (AUC: 92.8 vs 88.4). Statistical analysis of the ROC curves showed that there was a significant improvement of MetaPred2CS performance both over that of the Bayesian approach and STRING (*p-value* < 0.05).

## Conclusion

In this work we present a novel sequence-based prediction method designed specifically for TCS signalling networks: MetaPred2CS. The method was systematically assessed under different benchmarking scenarios, and performed well in all conditions, including using species-specific gene sets and TCS with different genome architecture features (i.e. neighbouring proteins vs. orphans). We show that integration of individual prediction methodologies improves the performance of the predictions, and that MetaPred2CS prediction performance compared favourably to existing methodologies. MetaPred2CS is accessible through a dedicated web-server at http://metapred2cs.ibers.aber.ac.uk.

## Additional files

Additional file 1:
**Information on P+, P-, NP+, OP+, SP+ and SP- datasets.** The experimental source of interaction data, organisms, relevant references and prediction scores of the MetaPred2CS, Bayesian method and STRING are provided. (XLSX 51 kb)
Additional file 2:
**Diagrammatic representation of P+, P-, NP+, OP+, SP+ and SP- datasets.** (PDF 76 kb)
Additional file 3:
**This file contains two supplementary tables.** Table S1 represents the content and organism distribution of the reference genome database and in Table S2 employed error cost (c) and gamma values (g) on MetaPred2CS under different K-fold cross validation values and combination of independent methods. (DOCX 23 kb)

## References

[1] Whitworth DE, Filloux A (2012). Two-component regulatory systems in prokaryotes. Bacterial Regulatory Networks.

[2] Appleby JL, Parkinson JS, Bourret RB (1996). Signal transduction via the multi-step phosphorelay: not necessarily a road less traveled. Cell.

[3] Whitworth DE, Gross R, Beier D (2012). Classification and organization of two-component systems. Two-component Systems in Bacteria.

[4] Ortet P, Whitworth DE, Santaella C, Achouak W, Barakat M (2015). P2CS: updates of the prokaryotic two-component systems database. Nucleic Acids Res.

[5] Barakat M, Ortet P, Whitworth DE (2013). P2RP: a Web-based framework for the identification and analysis of regulatory proteins in prokaryotic genomes. BMC Genomics.

[6] Ulrich LE, Zhulin IB (2010). The MiST2 database: a comprehensive genomics resource on microbial signal transduction. Nucleic Acids Res.

[7] Laub MT, Goulian M (2007). Specificity in two-component signal transduction pathways. Annu Rev Genet.

[8] Willett JW, Tiwari N, Müller S, Hummels KR, Houtman JCD, Fuentes EJ (2013). Specificity residues determine binding affinity for two-component signal transduction systems. mBio.

[9] Laub MT, Biondi EG, Skerker JM (2007). Phosphotransfer profiling: systematic mapping of two-component signal transduction pathways and phosphorelays. Methods Enzymol.

[10] Skerker JM, Prasol MS, Perchuk BS, Biondi EG, Laub MT (2005). Two-component signal transduction pathways regulating growth and cell cycle progression in a bacterium: a system-level analysis. PLoS Biol.

[11] Lee H-N, Jung K-E, Ko I-J, Baik HS, Oh J-I (2012). Protein-protein interactions between histidine kinases and response regulators of Mycobacterium tuberculosis H37Rv. J Microbiol Seoul Korea.

[12] Sato S, Shimoda Y, Muraki A, Kohara M, Nakamura Y, Tabata S (2007). A large-scale protein protein interaction analysis in Synechocystis sp. PCC6803. DNA Res Int J Rapid Publ Rep Genes Genomes.

[13] Shimoda Y, Shinpo S, Kohara M, Nakamura Y, Tabata S, Sato S (2008). A large scale analysis of protein-protein interactions in the nitrogen-fixing bacterium Mesorhizobium loti. DNA Res Int J Rapid Publ Rep Genes Genomes.

[14] Whitworth DE, Millard A, Hodgson DA, Hawkins PF (2008). Protein-protein interactions between two-component system transmitter and receiver domains of Myxococcus xanthus. Proteomics.

[15] Friedberg I, Harder T, Godzik A (2006). JAFA: a protein function annotation meta-server. Nucleic Acids Res.

[16] Ishida T, Kinoshita K (2008). Prediction of disordered regions in proteins based on the meta approach. Bioinforma Oxf Engl.

[17] Kurowski MA, Bujnicki JM (2003). GeneSilico protein structure prediction meta-server. Nucleic Acids Res.

[18] Pawlowski M, Gajda MJ, Matlak R, Bujnicki JM (2008). MetaMQAP: a meta-server for the quality assessment of protein models. BMC Bioinformatics.

[19] Saini HK, Fischer D (2005). Meta-DP: domain prediction meta-server. Bioinforma Oxf Engl.

[20] Xue B, Dunbrack RL, Williams RW, Dunker AK, Uversky VN (1804). PONDR-FIT: a meta-predictor of intrinsically disordered amino acids. Biochim Biophys Acta.

[21] Schlessinger A, Punta M, Yachdav G, Kajan L, Rost B (2009). Improved disorder prediction by combination of orthogonal approaches. PloS One.

[22] Needham CJ, Bradford JR, Bulpitt AJ, Westhead DR (2006). Inference in Bayesian networks. Nat Biotechnol.

[23] Segura J, Jones PF, Fernandez-Fuentes N (2012). A holistic in silico approach to predict functional sites in protein structures. Bioinforma Oxf Engl.

[24] Assi SA, Tanaka T, Rabbitts TH, Fernandez-Fuentes N (2010). PCRPi: Presaging Critical Residues in Protein interfaces, a new computational tool to chart hot spots in protein interfaces. Nucleic Acids Res.

[25] Noble WS (2006). What is a support vector machine?. Nat Biotechnol.

[26] Yang ZR (2004). Biological applications of support vector machines. Brief Bioinform.

[27] Pazos F, Valencia A (2002). In silico two-hybrid system for the selection of physically interacting protein pairs. Proteins.

[28] Pazos F, Valencia A (2001). Similarity of phylogenetic trees as indicator of protein-protein interaction. Protein Eng.

[29] Sun J, Xu J, Liu Z, Liu Q, Zhao A, Shi T (2005). Refined phylogenetic profiles method for predicting protein-protein interactions. Bioinforma Oxf Engl.

[30] Enright AJ, Iliopoulos I, Kyrpides NC, Ouzounis CA (1999). Protein interaction maps for complete genomes based on gene fusion events. Nature.

[31] Shoemaker BA, Panchenko AR (2007). Deciphering protein-protein interactions. Part II. Computational methods to predict protein and domain interaction partners. PLoS Comput Biol.

[32] Burger L, van Nimwegen E (2008). Accurate prediction of protein-protein interactions from sequence alignments using a Bayesian method. Mol Syst Biol.

[33] Von Mering C, Huynen M, Jaeggi D, Schmidt S, Bork P, Snel B (2003). STRING: a database of predicted functional associations between proteins. Nucleic Acids Res.

[34] Stark C, Breitkreutz B-J, Reguly T, Boucher L, Breitkreutz A, Tyers M (2006). BioGRID: a general repository for interaction datasets. Nucleic Acids Res.

[35] Xenarios I, Salwínski L, Duan XJ, Higney P, Kim S-M, Eisenberg D (2002). DIP, the Database of Interacting Proteins: a research tool for studying cellular networks of protein interactions. Nucleic Acids Res.

[36] Hermjakob H, Montecchi-Palazzi L, Lewington C, Mudali S, Kerrien S, Orchard S (2004). IntAct: an open source molecular interaction database. Nucleic Acids Res.

[37] Hermjakob H, Montecchi-Palazzi L, Bader G, Wojcik J, Salwinski L, Ceol A (2004). The HUPO PSI’s molecular interaction format--a community standard for the representation of protein interaction data. Nat Biotechnol.

[38] Magrane M, Consortium U (2011). UniProt Knowledgebase: a hub of integrated protein data. Database J Biol Databases Curation.

[39] Zanzoni A, Montecchi-Palazzi L, Quondam M, Ausiello G, Helmer-Citterich M, Cesareni G (2002). MINT: a Molecular INTeraction database. FEBS Lett.

[40] Cock PJA, Whitworth DE (2007). Evolution of gene overlaps: relative reading frame bias in prokaryotic two-component system genes. J Mol Evol.

[41] Cock PJA, Whitworth DE (2007). Evolution of prokaryotic two-component system signaling pathways: gene fusions and fissions. Mol Biol Evol.

[42] Williams RHN, Whitworth DE (2010). The genetic organisation of prokaryotic two-component system signalling pathways. BMC Genomics.

[43] Muley VY, Ranjan A (2012). Effect of reference genome selection on the performance of computational methods for genome-wide protein-protein interaction prediction. PloS One.

[44] Tatusova T, Ciufo S, Fedorov B, O’Neill K (2014). Tolstoy I.

[45] Moreno-Hagelsieb G, Collado-Vides J (2002). A powerful non-homology method for the prediction of operons in prokaryotes. Bioinformatics.

[46] Fernandez-Fuentes N, Rai BK, Madrid-Aliste CJ, Fajardo JE, Fiser A (2007). Comparative protein structure modeling by combining multiple templates and optimizing sequence-to-structure alignments. Bioinforma Oxf Engl.

[47] Sun J, Sun Y, Ding G, Liu Q, Wang C, He Y (2007). InPrePPI: an integrated evaluation method based on genomic context for predicting protein-protein interactions in prokaryotic genomes. BMC Bioinformatics.

[48] Bhagwat M, Aravind L (2007). PSI-BLAST tutorial. Methods Mol Biol Clifton NJ.

[49] Strong M, Mallick P, Pellegrini M, Thompson MJ, Eisenberg D (2003). Inference of protein function and protein linkages in Mycobacterium tuberculosis based on prokaryotic genome organization: a combined computational approach. Genome Biol.

[50] Ermolaeva MD, White O, Salzberg SL (2001). Prediction of operons in microbial genomes. Nucleic Acids Res.

[51] Smith TF, Waterman MS (1981). Identification of common molecular subsequences. J Mol Biol.

[52] Pearson WR, Misener S, Krawetz SA (1999). Flexible Sequence Similarity Searching with the FASTA3 Program Package. Bioinformatics Methods and Protocols.

[53] Chang C-C, Lin C-J (2011). LIBSVM: A Library for Support Vector Machines. ACM Trans Intell Syst Technol.

[54] Cho BH, Yu H, Lee J, Chee YJ, Kim IY, Kim SI (2008). Nonlinear support vector machine visualization for risk factor analysis using nomograms and localized radial basis function kernels. IEEE Trans Inf Technol Biomed Publ IEEE Eng Med Biol Soc.

[55] Baldi P, Brunak S, Chauvin Y, Andersen CAF, Nielsen H (2000). Assessing the accuracy of prediction algorithms for classification: an overview. Bioinformatics.

[56] Zweig MH, Campbell G (1993). Receiver-operating characteristic (ROC) plots: a fundamental evaluation tool in clinical medicine. Clin Chem.

[57] Vergara IA, Norambuena T, Ferrada E, Slater AW, Melo F (2008). StAR: a simple tool for the statistical comparison of ROC curves. BMC Bioinformatics.

[58] Zhang QC, Petrey D, Deng L, Qiang L, Shi Y, Thu CA (2012). Structure-based prediction of protein-protein interactions on a genome-wide scale. Nature.

[59] Planas-Iglesias J, Bonet J, García-García J, Marín-López MA, Feliu E, Oliva B (2013). Understanding protein-protein interactions using local structural features. J Mol Biol.

