# Supplementary material for: Genome-wide prediction of prokaryotic two-component system networks using a sequence-based meta-predictor
Source: BMC Bioinformatics. 2015 Sep 18;16:297. doi: 10.1186/s12859-015-0741-7 (PMC4575426; doi:10.1186/s12859-015-0741-7)
Supplement: Additional file 2: — Diagrammatic representation of P+, P-, NP+, OP+, SP+ and SP- datasets. (PDF 76 kb) [file 12859_2015_741_MOESM2_ESM.pdf]

Acronyms and definitions

- **P+:** Gold standard dataset interacting
- **P-:** Gold standard dataset of non-interacting
- **NP+:** Subset of P+ composed of encoded by neighbouring genes
- **OP+:** Subset of P+ composed of encoded by non-neighbouring genes
- **T:** The dataset compiled in Burger and van Nimwegen, Mol Sys Biol 2008
- **T+:** Interacting included in T dataset
- **T-:** Non-interacting included T dataset
- **SP+:** Subset of P+ dataset composed of present in Burger and van Nimwegen's database (also used to compare to STRING database)
- **SP-:** Subset of P+ dataset composed of present in Burger and van Nimwegen's database (also used to compare to STRING database)
- **Eco =** *Escherichia coli* K-12 MG1655
- **Sll =** *Synechocystis sp*. PCC6803
- **Mll =** *Mesorhizobium loti* MAFF303099
- **Mxan =** *Myxococcus xanthus* DK 1622

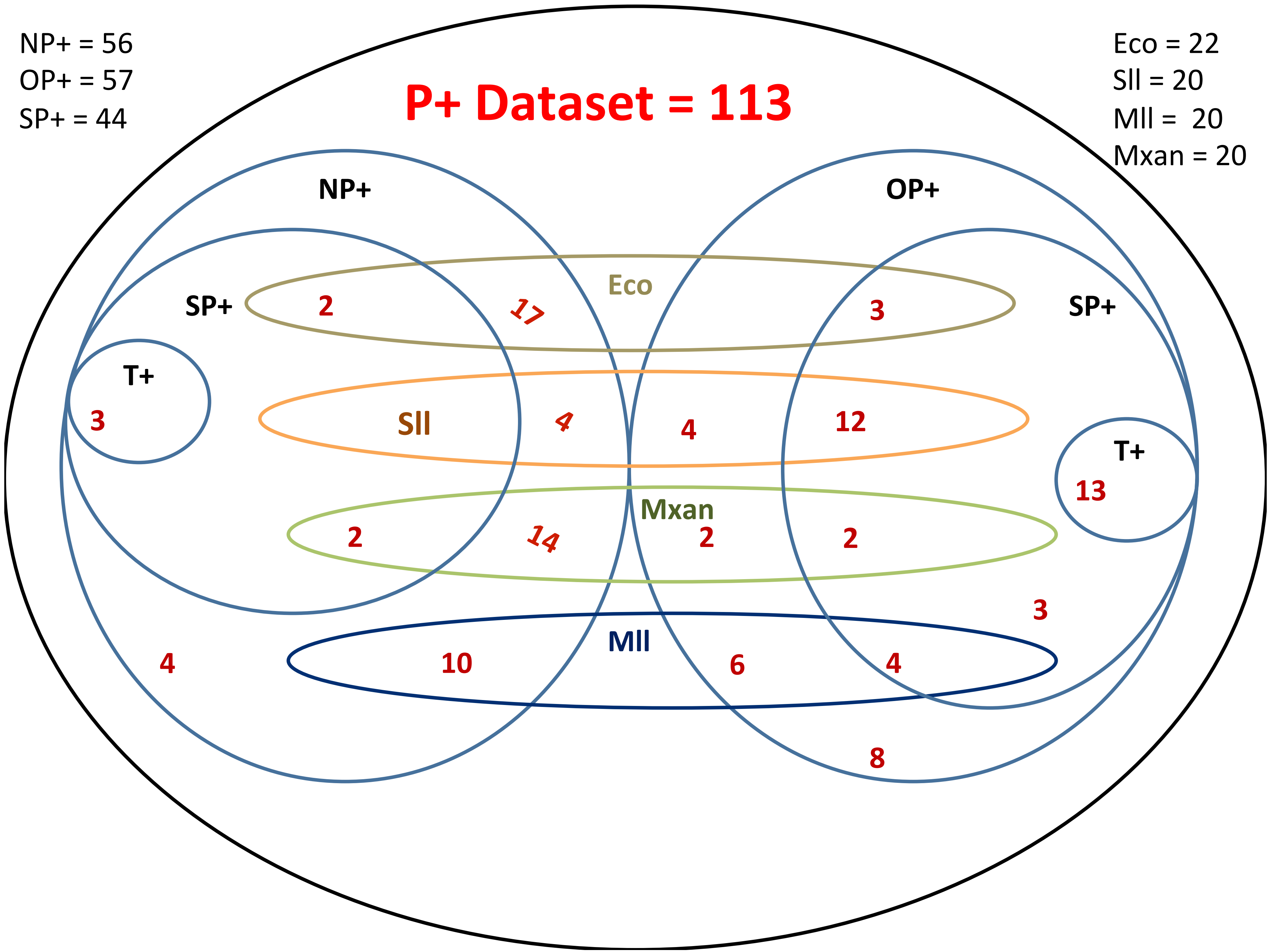
NP+ = 56
OP+ = 57
SP+ = 44
P+ Dataset = 113
Eco = 22
SII = 20
MII = 20
Mxan = 20
NP+
OP+
SP+
Eco
2
17
3
SP+
T+
3
SII
4
4
12
T+
13
Mxan
2
14
2
2
3
MII
4
10
6
4
8

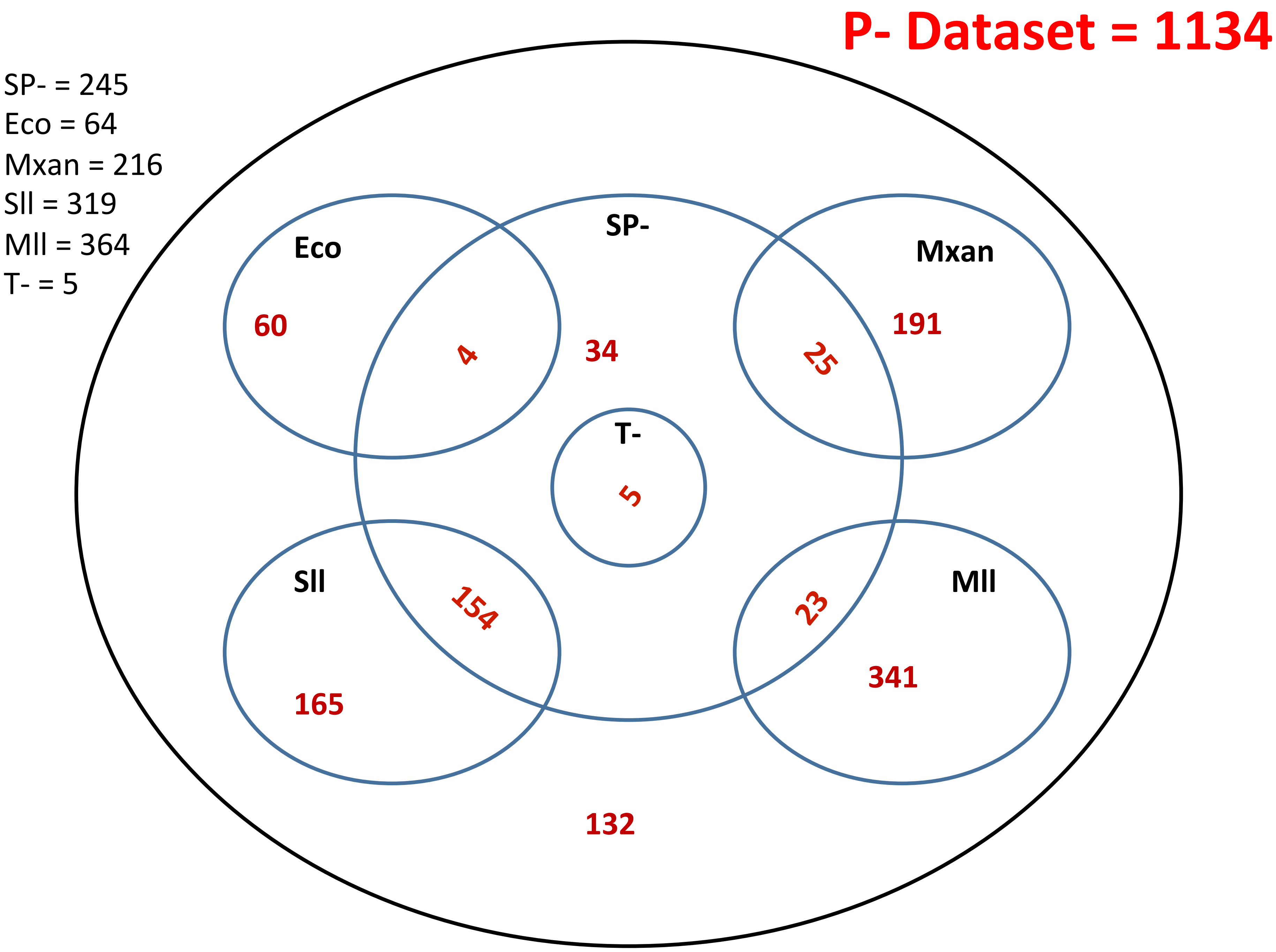
P- Dataset = 1134
SP- = 245
Eco = 64
Mxan = 216
SII = 319
MII = 364
T- = 5
Eco
SP-
Mxan
60
4
34
25
191
T-
5
SII
154
23
MII
341
165
132
