# Supplementary material for: Genome-wide prediction of prokaryotic two-component system networks using a sequence-based meta-predictor
Source: BMC Bioinformatics. 2015 Sep 18;16:297. doi: 10.1186/s12859-015-0741-7 (PMC4575426; doi:10.1186/s12859-015-0741-7)
Supplement: Additional file 3: — This file contains two supplementary tables. Table S1 represents the content and organism distribution of the reference genome database and in Table S2 employed error cost (c) and gamma values (g) on MetaPred2CS under different K-fold cross validation values and combination of independent methods. (DOCX 23 kb) [file 12859_2015_741_MOESM3_ESM.docx]

Additional file of ***“Genome-Wide Prediction of Prokaryotic Two-component System Networks using a Sequence-Based Meta-Predictor: MetaPred2CS”*** by Kara et al.

**Table S1.** Taxonomic distribution of the reference genome database.

|  |  | Class/Group | Number of organisms Included in database |
| --- | --- | --- | --- |
| 1 |  | Alphaproteobacteria | 27 |
| 2 |  | Aquificae | 1 |
| 3 |  | Firmicutes | 49 |
| 4 |  | Bacteroidetes/Chlorobi | 5 |
| 5 |  | Delta/epsilon subdivisions | 10 |
| 6 |  | Actinobacteria | 16 |
| 7 |  | Betaproteobacteria | 13 |
| 8 |  | Spirochaetes | 2 |
| 9 |  | Gammaproteobacteria | 49 |
| 10 |  | Chlamydiae/Verrucomicrobia | 10 |
| 11 |  | Deinococcus-Thermus | 3 |
| 12 |  | Fusobacteria | 1 |
| 13 |  | Cyanobacteria | 11 |
| 14 |  | Fibrobacteres/Acidobacteria | 3 |
| 15 |  | Spirochaetes | 4 |
| 16 |  | Tenericutes | 12 |
| 17 |  | Planctomycetes | 1 |
| 18 |  | Thermotogae | 1 |
| 19 |  | Chloroflexi | 2 |
| 20 |  | Crenarchaeota | 6 |
| 21 |  | Euryarchaeota | 16 |
| 22 |  | Nanoarchaeota | 1 |

**Table S2.** Error cost (c) and gamma values (g) employed by MetaPred2CS under different K-fold cross validation values and combinations of independent methods. **1:** excludes i2h method, **2:** excludes MT method, **3:** excludes GF method, **4:** excludes PP method, **5:** excludes GN method, **6:** excludes GO method, **7**: excludes GN and GO methods, **8:** includes all six methods. In table *c* and *g* values represents the error cost and gamma values, respectively. The final classifier was selected as the 8^th^ combinations (all methods) according to 10-fold cross-validation performance (values represented in bold).

|  | SVM Parameters According to Cross-validation Level | | | | | | Performance of Classifiers According to Cross-validation Level | | | | | |
| --- | --- | --- | --- | --- | --- | --- | --- | --- | --- | --- | --- | --- |
|  | **5-fold** | | **10-fold** | | **20-fold** | | **5-fold** | | **10-fold** | | **20-fold** | |
|  | ***c*** | ***g*** | ***c*** | ***g*** | ***c*** | ***g*** | **AUC** | **MCC** | **AUC** | **MCC** | **AUC** | **MCC** |
| 1 | 16.0 | 1.0 | 32.0 | 0.25 | 16.0 | 0.25 | 88.87 | 0.401 | 88.86 | 0.401 | 89.19 | 0.403 |
| 2 | 16.0 | 0.25 | 16.0 | 0.25 | 32.0 | 0.125 | 94.11 | 0.455 | 94.69 | 0.500 | 94.68 | 0.499 |
| 3 | 32.0 | 0.25 | 32.0 | 0.25 | 32.0 | 0.25 | 93.36 | 0.445 | 94.45 | 0.484 | 94.30 | 0.476 |
| 4 | 32.0 | 0.25 | 32.0 | 0.25 | 32.0 | 0.25 | 92.17 | 0.424 | 91.89 | 0.414 | 91.76 | 0.412 |
| 5 | 32.0 | 0.125 | 16.0 | 0.25 | 32.0 | 0.125 | 93.26 | 0.442 | 94.04 | 0.454 | 93.81 | 0.449 |
| 6 | 32.0 | 0.25 | 32.0 | 0.25 | 32.0 | 0.25 | 94.25 | 0.465 | 94.76 | 0.504 | 94.69 | 0.500 |
| 7 | 16.0 | 0.25 | 32.0 | 0.25 | 32.0 | 0.125 | 89.83 | 0.406 | 90.15 | 0.408 | 89.88 | 0.407 |
| 8 | 32.0 | 0.25 | **32.0** | **0.25** | 32.0 | 0.25 | 94.26 | 0.465 | **94.79** | **0.508** | 94.70 | 0.500 |
